# *Erratum:* Vol. 65, No. 50-51

**DOI:** 10.15585/mmwr.mm6602a9

**Published:** 2017-01-20

In the report “Outbreak of *Salmonella* Oslo Infections Linked to Persian Cucumbers — United States, 2016,” the footnote on page 1430 should have read “^†^ https://www.cdc.gov/foodnet/surveys/population.html**.”**

